# Trends in Imaging for Suspected Pulmonary Embolism Across US Health Care Systems, 2004 to 2016

**DOI:** 10.1001/jamanetworkopen.2020.26930

**Published:** 2020-11-20

**Authors:** Ralph C. Wang, Diana L. Miglioretti, Emily C. Marlow, Marilyn L. Kwan, May K. Theis, Erin J. A. Bowles, Robert T. Greenlee, Alanna K. Rahm, Natasha K. Stout, Sheila Weinmann, Rebecca Smith-Bindman

**Affiliations:** 1 Department of Emergency Medicine, University of California, San Francisco; 2 Department of Public Health Sciences, University of California, Davis; 3 Comprehensive Cancer Center, University of California, Davis; 4 Kaiser Permanente Washington Health Research Institute, Kaiser Permanente Washington, Seattle; 5 Department of Public Health Sciences, University of California, Davis; 6 Division of Research, Kaiser Permanente Northern California, Oakland; 7 Marshfield Clinic Research Institute, Marshfield Clinic Health System, Marshfield, Wisconsin; 8 Genomic Medicine Institute, Geisinger, Danville, Pennsylvania; 9 Massachusetts Department of Population Medicine, Harvard Medical School and Harvard Pilgrim Health Care Institute, Boston; 10 now with Center for Health Research, Kaiser Permanente Northwest, Portland, Oregon; 11 Center for Integrated Health Research, Kaiser Permanente Hawaii, Honolulu; 12 Department of Radiology and Biomedical Imaging, University of California, San Francisco; 13 Department of Epidemiology and Biostatistics, University of California, San Francisco; 14 Philip R. Lee Institute for Health Policy Studies, University of California, San Francisco

## Abstract

**Importance:**

In response to calls to reduce unnecessary diagnostic testing with computed tomographic pulmonary angiography (CTPA) for suspected pulmonary embolism (PE), there have been growing efforts to create and implement decision rules for PE testing. It is unclear if the use of advanced imaging tests for PE has diminished over time.

**Objective:**

To assess the use of advanced imaging tests, including chest computed tomography (CT) (ie, all chest CT except for CTPA), CTPA, and ventilation-perfusion (V/Q) scan, for PE from 2004 to 2016.

**Design, Setting, and Participants:**

Cohort study of adults by age group (18-64 years and ≥65 years) enrolled in 7 US integrated and mixed-model health care systems. Joinpoint regression analysis was used to identify years with statistically significant changes in imaging rates and to calculate average annual percentage change (growth) from 2004 to 2007, 2008 to 2011, and 2012 to 2016. Analyses were conducted between June 11, 2019, and March 18, 2020.

**Main Outcomes and Measures:**

Rates of chest CT, CTPA, and V/Q scan by year and age, as well as annual change in rates over time.

**Results:**

Overall, 3.6 to 4.8 million enrollees were included each year of the study, for a total of 52 343 517 person-years of follow-up data. Adults aged 18 to 64 years accounted for 42 223 712 person-years (80.7%) and those 65 years or older accounted for 10 119 805 person-years (19.3%). Female enrollees accounted for 27 712 571 person-years (52.9%). From 2004 and 2016, chest CT use increased by 66.3% (average annual growth, 4.4% per year), CTPA use increased by 450.0% (average annual growth, 16.3% per year), and V/Q scan use decreased by 47.1% (decreasing by 4.9% per year). The use of CTPA increased most rapidly from 2004 to 2006 (44.6% in those aged 18-64 years and 43.9% in those ≥65 years), with ongoing rapid growth from 2006 to 2010 (annual growth, 19.8% in those aged 18-64 years and 18.3% in those ≥65 years) and persistent but slower growth in the most recent years (annual growth, 4.3% in those aged 18-64 years and 3.0% in those ≥65 years from 2010 to 2016). The use of V/Q scanning decreased steadily since 2004.

**Conclusions and Relevance:**

From 2004 to 2016, rates of chest CT and CTPA for suspected PE continued to increase among adults but at a slower pace in more contemporary years. Efforts to combat overuse have not been completely successful as reflected by ongoing growth, rather than decline, of chest CT use. Whether the observed imaging use was appropriate or was associated with improved patient outcomes is unknown.

## Key Points

**Question:**

Given increasing concerns of imaging overuse for pulmonary embolism, has the use of computed tomographic pulmonary angiography (CTPA) decreased over time?

**Findings:**

Among 52 343 517 person-years of follow-up data in this cohort study, CTPA use increased during all periods, most rapidly from 2004 to 2006, with ongoing rapid growth during the next period (2006 to 2010), and persistent but slower growth in the most recent years (2010 to 2016). The use of ventilation-perfusion scanning decreased steadily since 2004.

**Meaning:**

From 2004 to 2016 in 7 US integrated and mixed-model health care systems, rates of CTPA use continued to increase among adults, raising questions about the effectiveness of efforts to reduce overuse.

## Teaser

This cohort study assesses the use of advanced imaging tests, including chest computed tomography, computed tomographic pulmonary angiography, and ventilation-perfusion scan, for pulmonary embolism from 2004 to 2016.

## Introduction

Venous thromboembolism is a common and potentially fatal disease, with an estimated lifetime prevalence of up to 5%.^1^ Approximately 20% of individuals with pulmonary embolism (PE) die before diagnosis or on the first day after their diagnosis.^1,2^ Because the signs and symptoms of PE are often nonspecific, advanced imaging is commonly used for the diagnosis.^2^ Ventilation-perfusion lung studies (V/Q scans, a nuclear medicine examination) were traditionally the noninvasive imaging test of choice, but in the early 2000s this imaging modality was supplanted by chest computed tomographic pulmonary angiography (CTPA).^3,4^ Chest CTPA is faster and more sensitive than V/Q scan; however, the improved sensitivity is at least in part believed to be owing to the detection of inconsequential, subsegmental PE.^4^

Since its introduction, CTPA has been embraced by emergency department (ED) and hospital physicians, and rates of use have increased dramatically, resulting in concerns of overuse.^5,6^ Previous studies examining trends in the use of computed tomography (CT) (based on survey or claims data) have not distinguished chest CT (ie, all chest CT except for CTPA) from CTPA. In 2001, CT (including CTPA) was used in 2.6% of ED visits for chest pain or shortness of breath, which increased to 12.5% in 2009, with an average growth of 28.1% per year.^7^ In an analysis of Medicare beneficiaries with suspected PE^8^ from 2002 to 2009, chest CT use increased 5-fold, but positivity rates (yield) decreased from 7.3% in 2002 to 5.9% in 2009. This finding suggests that a smaller percentage of patients have received the potential benefit of CTPA with respect to improved detection, and more patients have experienced potential harms, including exposure to ionizing radiation, intravenous contrast,^9,10^ and overdiagnosis. This observation is further supported by an increasing incidence of PE, with a lower case mortality rate but no change in overall PE mortality.^10,11,12^

In response to calls to curb unnecessary and wasteful diagnostic testing, there have been growing efforts to create and implement decision rules for PE testing that rely on risk stratification algorithms to reduce its unnecessary use. The Wells criteria (combined with D-dimer testing) and the PE rule-out criteria (PERC rule) have been derived, extensively validated, and implemented into clinical practice to identify low-risk patients for whom CTPA can be safely avoided.^2,4,13,14,15^ These risk stratification–based strategies were broadly disseminated through national educational campaigns, such as Choosing Wisely. Five societies (American College of Emergency Physicians, American College of Chest Physicians, American Thoracic Society, Society of Nuclear and Molecular Imaging, and American College of Radiology) published guidelines between 2012 and 2014 promoting the avoidance of CTPA for patients with low probability of PE and a negative D-dimer test result or who are PERC negative.^16,17^ The Centers for Medicare & Medicaid Services has also mandated the implementation of these clinical decision rules for Medicare beneficiaries by the creation of clinical decision support tools embedded in the electronic health record at the point of order entry to guide clinicians through risk stratification.^18,19^

The implications of these rules for diagnostic imaging use in actual practice is not known.^18,20,21^ Implementation studies^22,23^ of clinical decision support and clinician feedback have shown improved clinician adherence to guidelines but no reduction in CTPA use, and it is unclear if the use of advanced imaging tests for PE has diminished over time. Prior studies^7,8^ have examined CTPA use but were limited to single departments or institutions or did not distinguish chest CTPA from other chest CT or did not cover periods after Choosing Wisely. We assessed the use of chest CT, CTPA, and V/Q scan within 7 US health care systems (including ED, inpatient, and outpatient settings) from 2004 to 2016 to provide a robust assessment of diagnostic imaging use for PE over time.

## Methods

### Data Sources

In this retrospective cohort study, imaging use was obtained from 2004 to 2016 for individuals enrolled in the following 7 US integrated and mixed-model health care systems: Kaiser Permanente (KP) Hawaii, KP Northern California, KP Northwest (Oregon and Southwest Washington), KP Washington, Geisinger (Pennsylvania), Harvard Pilgrim Health Care Institute (New England), and Marshfield Clinic Health System (Wisconsin). These health care systems are members of the Health Care Systems Research Network^24^ and reflect fully integrated and mixed-model health care systems. All sites have available electronic health care information stored in a virtual data warehouse, including comprehensive capture of all imaging among enrollees.^25,26^ Imaging is captured using clinical and administrative data sources, including imaging done within and outside the health care system. The institutional review boards of all collaborating institutions approved the study, and a waiver of individual informed consent was obtained because of the use of deidentified data. The Strengthening the Reporting of Observational Studies in Epidemiology (STROBE) reporting guideline was followed.

### Imaging Use

For each calendar year of the study, individuals who initially enrolled, were continuously enrolled, or died during that year were included. Imaging examinations were coded using a combination of *Current Procedural Terminology*,^27^ *International Classification of Diseases*, *Ninth Revision*,^28^ *International Statistical Classification of Diseases*, *Tenth Revision*,^29^ and Health Care Financing Administration Common Procedural Coding System^30^ billing codes. Examinations were included regardless of the physician specialty billing for the study. We updated a previously used map of billing codes to anatomic area and imaging modality to identify chest CT, chest CTPA, and V/Q nuclear medicine examinations.^31^ Within these modalities, chest CTPA for PE was distinguished from chest non-CTPA scans (chest CT) using specific billing codes for PE. There are a total of 117 individual codes (83 CT chest, 9 CTPA, and 25 V/Q scan codes) used to identify chest CT, chest CTPA, and V/Q nuclear medicine examinations. Both professional and technical billing claims were used to capture use; however, to avoid overcounting, only a single imaging examination per person per day was included.

### Statistical Analysis

Analyses were conducted between June 11, 2019, and March 18, 2020, and we used all available data from each health care system. Analyses were stratified by imaging test and age group (18-64 years and ≥65 years) and sex (female vs male). We also accounted for follow-up time for individuals based on enrollment for that calendar year. Use rates were modeled with overdispersed Poisson regression, including main effects for examination year and contributing health care system. Absolute annual rates per 1000 person-years with 95% CIs were estimated from these overdispersed Poisson regression models. Rates were averaged across health care systems using equal weights. Relative rate comparisons were made within each period group (2007 vs 2004, 2011 vs 2008, and 2016 vs 2012). All analyses were based on study participants with complete imaging data. If individuals were missing imaging data, they were not included in the analytic cohort.

We used joinpoint regression analysis^32,33^ to identify years with statistically significant changes in imaging trends over time and to calculate the mean annual percentage change (growth) within each period and age group. The number of examinations per person-year and SEs estimated from the Poisson regression model were added into the Joinpoint software. A permutation test was used to identify the optimal number of change points for each group (imaging test and age), applying a Bonferroni correction to the type I error to correct for multiple testing. A maximum of 2 change points was allowed based on Joinpoint recommendations given the number of years included. Annual percentage changes and 95% CIs were estimated assuming that the rates change at a constant percentage every year on a log scale. A second approach also used joinpoint regression but using fixed, specified periods (2004-2007, 2008-2011, and 2012-2016) to generate mean annual percentage change (growth) within each period by imaging test and age group, allowing easier reporting in tabular format and comparisons across period age group. Poisson regression analyses were conducted using SAS, version 9.4 (SAS Institute Inc), and joinpoint regression was performed using Joinpoint Regression Program, version 4.7.0.0 (National Cancer Institute).^34^

## Results

Overall, 3.6 to 4.8 million enrollees were included in each year of the study, for a total of 52 343 517 person-years of follow-up data (Table 1). Adults aged 18 to 64 years accounted for 42 223 712 person-years (80.7%), and those 65 years or older accounted for 10 119 805 person-years (19.3%). Female enrollees accounted for 27 712 571 person-years (52.9%), and male enrollees accounted for 24 630 946 person-years (47.1%). Overall, patients underwent 1.8 million chest CT scans, 348 505 CTPA examinations (16.5% of all of the CTs performed in the chest), and 59 208 V/Q scans. Among the 7 health systems, the rate of chest CT ranged from 29.3 to 50.6 per 1000 person-years, the rate of CTPA ranged from 5.3 to 8.6 per 1000 person-years, and the rate of V/Q scans ranged from 0.8 to 1.8 per 1000 person-years across years. Chest CT scan rates were higher than CTPA examination rates (on average, 4 to 8 times higher across health systems), and both were higher than V/Q scan rates. Averaging across the entire study period, imaging rates for each of the 3 tests were approximately 4 times higher in adults 65 years or older compared with adults aged 18 to 64 years.

**Table 1.** Characteristics of the Study Population

Variable	No. of person-years	Chest CT		CTPA		V/Q scan	
		No. of examinations	Rate per 1000 person-years	No. of examinations	Rate per 1000 person-years	No. of examinations	Rate per 1000 person-years
Total No. of person-years	52 343 517	1 761 444	NA	348 505	NA	59 208	NA
US site							
1	1 527 023	67 011	43.9	8427	5.5	2781	1.8
2	2 239 325	113 222	50.6	11 975	5.3	3615	1.6
3	1 966 518	67 613	34.4	10 958	5.6	1525	0.8
4	4 354 227	154 240	35.4	37 416	8.6	5523	1.3
5	5 258 195	199 503	37.9	40 275	7.7	5902	1.1
6	6 881 144	278 419	40.5	42 532	6.2	6254	0.9
7	30 117 085	881 436	29.3	196 922	6.5	33 608	1.1
Age							
Adults 18-64 y	42 223 712	878 972	20.8	180 899	4.3	23 843	0.6
Adults ≥65 y	10 119 805	882 472	87.2	167 606	16.6	35 365	3.5
Sex							
Female	27 712 571	939 973	33.9	201 935	7.3	34 460	1.2
Male	24 630 946	821 471	33.4	146 570	6.0	24 748	1.0
Calendar year							
2004	3 689 169	91 978	24.9	6604	1.8	6383	1.7
2005	3 680 738	104 072	28.3	10 240	2.8	5362	1.5
2006	3 719 631	116 961	31.4	14 557	3.9	4393	1.2
2007	3 732 516	120 893	32.4	17 005	4.6	4296	1.2
2008	3 816 722	116 790	30.6	19 826	5.2	4096	1.1
2009	3 805 571	125 734	33.0	25 222	6.6	4216	1.1
2010	3 883 048	128 089	33.0	27 837	7.2	4124	1.1
2011	4 030 961	134 743	33.4	32 483	8.1	4364	1.1
2012	4 155 719	139 622	33.6	34 232	8.2	4585	1.1
2013	4 184 375	144 447	34.5	34 609	8.3	4488	1.1
2014	4 288 416	159 570	37.2	36 593	8.5	4287	1.0
2015	4 510 752	177 721	39.4	41 476	9.2	4200	0.9
2016	4 845 899	200 824	41.4	47 821	9.9	4414	0.9

Abbreviations: Chest CT, all chest computed tomography except for CTPA; CTPA, computed tomographic pulmonary angiography for pulmonary embolism; NA, not applicable; V/Q scan, ventilation-perfusion scan for pulmonary embolism.

Imaging with chest CT and CTPA increased over time, whereas V/Q scan use decreased over time (Table 1). From 2004 and 2016, chest CT use increased by 66.3% (average annual growth, 4.4% per year, increasing from 24.9 to 41.4 per 1000 person-years), CTPA use increased by 450.0% (average annual growth, 16.3% per year, increasing from 1.8 to 9.9 per 1000 person-years, and V/Q scan use decreased by 47.1% (decreasing by 4.9% per year, decreasing from 1.7 to 0.9 per 1000 person-years). In 2004, imaging rates of CTPA and V/Q scanning were approximately equal across both age groups (4.0 and 4.8 per 1000 person-years for CTPA and V/Q scans, respectively, in adults 65 years or older and 1.1 per 1000 person-years for both CTPA and V/Q scans in adults aged 18-64 years). By 2016, CTPA was 7.4 times as common as V/Q scanning in adults 65 years or older (20.1 vs 2.7 per 1000 person-years) and 15.5 times as common as V/Q scanning in adults aged 18 to 64 years (6.2 vs 0.4 per 1000 person-years). Therefore, the growth in use of CTPA exceeded the decline in V/Q scanning.

**Figure 1.**
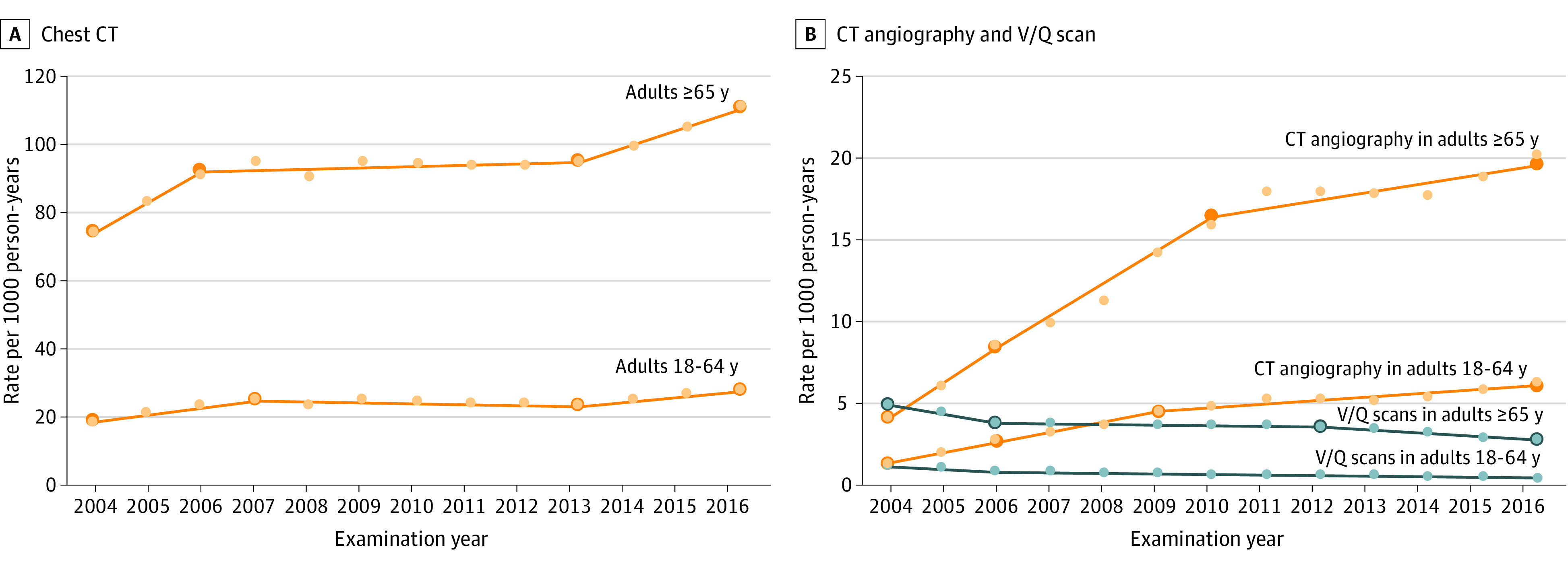
Chest Imaging Rates per 1000 Person-Years by Imaging Test and Age Group, With Annual Percentage Changes in Rates Based on Joinpoint Regression Analysis A and B, Small circles represent raw estimates of rates of imaging use for each calendar year. Superimposed lines are regression lines obtained from joinpoint regression analysis. Large circles represent the change points identified by joinpoint regression analysis as statistically significant changes in the annual growth rate. Chest CT indicates all chest computed tomography except for chest computed tomographic pulmonary angiography for pulmonary embolism. V/Q scan indicates ventilation-perfusion scan for pulmonary embolism.

The annual rates of chest CT, CTPA, and V/Q scan use over time for adults aged 18 to 64 years and adults 65 years or older based on joinpoint regression analysis are shown in Figure 1. For chest CT and CTPA, the patterns of change over time were similar for both age groups. For adults aged 18 to 64 years, chest CT had rapid growth from 2004 to 2007 (10.0% annual growth), no growth from 2007 to 2013 (−0.9% annual growth), and reacceleration in growth from 2013 to 2016 (5.8% annual growth). Similarly, for adults 65 years or older, chest CT had rapid growth from 2004 to 2006 (11.6% annual growth), followed by little growth from 2006 to 2013 (0.4% annual growth), and reacceleration in growth from 2013 to 2016 (5.4% annual growth). The use of CTPA increased over the study period in adults aged 18 to 64 years, although growth rates declined over time. For example, the use of CTPA increased most rapidly from 2004 to 2006 (44.6% in those aged 18-64 years and 43.9% in those ≥65 years), with ongoing rapid growth from 2006 to 2010 (annual growth, 19.8% in those aged 18-64 years and 18.3% in those ≥65 years) and persistent but slower growth in the most recent years (annual growth, 4.3% in those aged 18-64 years and 3.0% in those ≥65 years from 2010 to 2016). Similar patterns were observed for CTPA use among adults 65 years or older. The use of V/Q scanning decreased steadily since 2004 (annual decline across the different segments ranged from 0.9% to 12.9%).

The average growth using fixed periods is summarized in Table 2. Overall, the annual change in chest CT, CTPA, and V/Q scan use was similar between adults aged 18 to 64 years and adults 65 years or older. From 2004 to 2007, the annual change in chest CT was 7.8% in adults aged 18 to 64 years and 10.0% in adults 65 years or older. From 2008 to 2011, the annual change decreased in both age groups; from 2012 to 2016, the annual change increased to 4.1% in both age groups. The use of CTPA increased most rapidly in the earliest period, and use continued to increase at a slower rate in the subsequent periods for both age groups. The annual decrease in V/Q scan use was most rapid in the earliest period, but use continued to decrease in subsequent periods for both age groups.

**Table 2.** Annual Change in Imaging Use by Imaging Test, Stratified by Age Group and Sex

Variable	Annual change from prior period, % (95% CI)		
	Chest CT	Chest CTPA	V/Q scan
**Adults 18-64 y**			
2004-2007	10.0 (4.4 to 15.9)	35.8 (17.2 to 57.4)	−15.5 (−20.5 to −10.3)
2008-2011	−0.9 (−3.0 to 1.2)	9.3 (4.9 to 13.7)	−6.0 (−7.5 to −4.5)
2012-2016	4.1 (1.6 to 6.7)	4.3 (2.4 to 6.3)	−6.0 (−7.5 to −4.5)
**Adults ≥65 y**			
2004-2007	7.8 (4.2 to 11.4)	34.8 (15.3 to 57.6)	−9.1 (−12.4 to −5.6)
2008-2011	0.4 (−0.3 to 1.2)	13.0 (7.7 to 18.5)	−0.9 (−2.4 to 0.5)
2012-2016	4.1 (2.7 to 5.5)	3.0 (0.8 to 5.3)	−6.2 (−8.1 to −4.2)
**Female sex**			
2004-2007	7.8 (4.2 to 11.4)	34.8 (15.3 to 57.6)	−9.1 (−12.4 to −5.6)
2008-2011	0.4 (−0.3 to 1.2)	13.0 (7.7 to 18.5)	−0.9 (−2.4 to 0.5)
2012-2016	4.1 (2.7 to 5.5)	3.0 (0.8 to 5.3)	−6.2 (−8.1 to −4.2)
**Male sex**			
2004-2007	10.0 (4.4 to 15.9)	35.8 (17.2 to 57.4)	−15.5 (−20.5 to −10.3)
2008-2011	−0.9 (−3.0 to 1.2)	9.3 (4.9 to 13.7)	−6.0 (−7.5 to −4.5)
2012-2016	4.1 (1.6 to 6.7)	4.3 (2.4 to 6.3)	−6.0 (−7.5 to −4.5)

Abbreviations: Chest CT, all chest computed tomography except for CTPA; chest CTPA, computed tomographic pulmonary angiography for pulmonary embolism; V/Q scan, ventilation-perfusion scan for pulmonary embolism.

**Figure 2.**
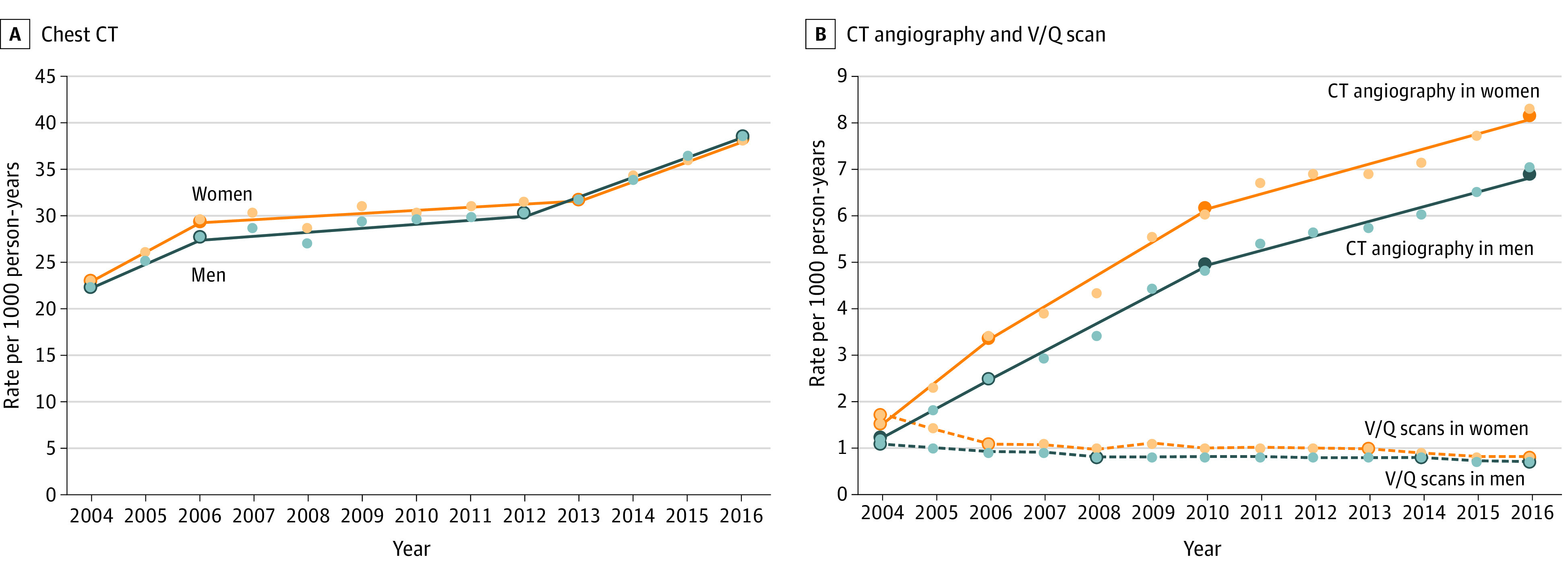
Chest Imaging Rates per 1000 Person-Years by Imaging Test and Sex, With Annual Percentage Changes in Rates Based on Joinpoint Regression Analysis A and B, Small circles represent raw estimates of rates of imaging use for each calendar year. Superimposed lines are regression lines obtained from joinpoint regression analysis. Large circles represent the change points identified by joinpoint regression analysis as statistically significant changes in the annual growth rate. Chest CT indicates all chest computed tomography except for CTPA; and V/Q scan, ventilation-perfusion scan for pulmonary embolism.

The annual rates of chest CT, CTPA, and V/Q use over time for female and male participants based on joinpoint regression analysis are shown in Figure 2. The use of chest CT increased from 2004 to 2006, plateaued between 2006 and 2012, and increased again from 2012 to 2016. For chest CT and CTPA, the patterns of change over time were similar for both groups. The use of CTPA increased over the study period, although growth rates declined over time. The use of V/Q scanning decreased steadily since 2004.

## Discussion

The use of CTPA for suspected PE across 7 US integrated health care systems has continued to increase in recent years. Annual growth in CTPA was highest in the earlier years of the study (ie, from 2004 to 2006), but imaging with CTPA has continued to increase in both adults aged 18 to 64 years and adults 65 years or older. In contrast, nuclear medicine imaging had a consistent decline in both age categories and across all health care systems. However, the growth in CTPA far outpaced the decline in V/Q scanning.

Previous studies^35,36^ of diagnostic imaging use in large US health care systems have found that older patients have higher rates of advanced imaging overall compared with younger patients. Imaging use increased steeply with age for CT, nuclear medicine, and magnetic resonance imaging, but in particular for CT scan.^35,36^ In the previous study^36^ of imaging use across the same 7 US integrated and mixed-model health care systems, CT imaging rates per person-years were highest in adults 65 years or older across most anatomic regions. Findings in the present study demonstrate a similar pattern for the use of CTPA to screen for PE. The exact reasons for the higher rates of imaging in adults 65 years or older are unclear but are likely multifactorial. It is well known that age is a risk factor for both PE and mortality in those diagnosed as having PE. This risk is reflected in clinical decision rules, such as the PERC rule, in which age 50 years or older is a risk factor that prevents exclusion of PE on clinical grounds. D-dimer testing is used to screen for PE and is more likely to be elevated in older patients than in younger patients. In addition, although cancer risk from radiation is often thought to decline with age, models suggest that cancer risk declines with age until middle age, when cancer risk may then increase in a U-shaped distribution.^37,38^ Therefore, radiation-related cancer risk after exposure in middle and older ages may be higher than previously believed.

Prior studies^7,8,39^ have examined the use of CT scan for suspected PE over time. Feng et al^7^ analyzed results of the National Hospital Ambulatory Medical Care Survey (NHAMCS) from 2001 to 2009, finding rapid increases in CT use during that period among patients seen in the ED for chest pain and shortness of breath. Similarly, Kocher et al^39^ analyzed the 1996 to 2007 NHAMCS and found large increases in CT use over time. However, these studies^7,8,39^ were limited by a lack of granularity surrounding imaging type, with the NHAMCS not differentiating between CT scan imaging types or anatomic regions. The NHAMCS also does not collect data regarding V/Q scan use. Furthermore, the NHAMCS data are limited to ED care. Venkatesh et al^8^ used Medicare analytic files to evaluate CT use to screen for PE among Medicare beneficiaries presenting to the ED between 2000 and 2009. The investigators reported steadily increasing rates of CT use, although their analysis was limited to a specific age range (in most cases, adults 65 years or older). Results in the present study reflect imaging from 2004 through 2016 among adults of all ages at 7 health care systems across the United States, and we were able to differentiate the use of CTPA from the use of chest CT, as well as assess the use of V/Q scan.

Both CT scan and CT angiography are valuable diagnostic tests that in many cases has led to accurate diagnoses and improved patient outcomes.^2^ However, 1 in 4 Americans receive CT scans each year, and many of these tests are unnecessary and expose patients to a number of risks, including anxiety and discomfort, ionizing radiation, and incidental findings.^10^ Exposure to radiation is believed to increase the lifetime risk of cancer,^10^ and the National Cancer Institute has estimated that 2% of cancers are iatrogenic.^9^ Incidental findings often lead to additional testing and unnecessary procedures. A study^40^ of Medicare beneficiaries found a statistically significant association between the number of CT scans of the abdomen or pelvis and the performance of nephrectomy. There are a number of reasons for the increase in CT use over time. The technology of CT scanning continues to improve, resulting in faster, more accurate studies. Clinicians increasingly rely on CT scans to avoid missing serious conditions and incurring malpractice claims. Payment models have incentivized clinicians and health systems to perform imaging, with few disincentives to request low-yield or inappropriate studies.

Several studies have found that well-conducted and validated approaches to reduce CTPA overuse are not having the desired impact.^2,14,15^ As demonstrated in the present study, not only have CTPA imaging rates not declined, but they have also continued to show 3.0% to 4.3% annual growth through 2016. The Wells criteria and D-dimer testing strategies have been modified over the last 2 decades, reformulated from 3 levels of risk (low, moderate, and high) to 2 levels (unlikely vs likely) to simplify decision-making at the bedside.^41^ D-dimer cutoffs have been altered to include age adjustment to better identify older patients who are at low risk.^14^ The YEARS study algorithm was derived and validated to produce a decision rule for suspected PE, with few items to simplify score calculation.^42^ Despite efforts and national initiatives to disseminate and implement these research findings,^2,14,15,42^ we found that CTPA use to screen for PE continues to increase. It is not clear why such efforts have not curtailed the growth in CTPA imaging, except to note that contributors to CT use persist, such as fear of missing PE, concerns about malpractice,^43^ improvements in technology and CT availability, and financial incentives. Also, it may be that approaches to developing risk stratification tools have been overly conservative, and the goal to create decision tools with high sensitivity may render such tools inefficient in that they may not identify a large proportion of low-risk patients in whom testing can be deferred. The Wells criteria for suspected PE contain a heavily weighted subjective component, which although providing flexibility might be interpreted in an overly cautious manner by clinicians. Finally, studies of the implementation of these clinical decision rules have not been conducted using optimal randomized designs, resulting in an inability to rigorously measure their impact on imaging overuse.

These observations suggest that the process of incorporating imaging tests into clinical practice should be governed by rigorous analysis of the benefits and harms for patients and health systems. The use of CTPA was rapidly embraced as the preferred first-line diagnostic test for suspected PE soon after reports touted its greater sensitivity than V/Q scan^4^ but before other outcomes were assessed, such as the rate of incidental diagnoses and their potential for overtreatment, as well as before the prognosis of subsegmental thrombi was understood.^6,11^ Furthermore, the ready availability of CT scanning likely promotes its use as a first-line test over others, such as the V/Q scan. Therefore, the continued use of CTPA as the first-line imaging test for PE may not have been guided by a balanced consideration of all benefits and harms. However, as shown by the results herein, deimplementation of tests once adopted is difficult, even when there is widespread agreement that the tests are overused.

### Limitations

This study has several limitations. First, the study included US patients enrolled in a limited number of health care systems, all of which used health maintenance organization models of care either in part or in whole. These health care systems were chosen because they are members of the National Cancer Institute–supported Health Care Systems Research Network and collect data in a common format that are locally stored in virtual data warehouses. These systems are diverse; however, results in the present study are limited because all of the included sites are integrated health care systems that used health maintenance organization models of care either in part or in whole. Patterns of imaging over time among these patients may not represent patterns among individuals covered by fee-for-service plans with different incentives and disincentives. For example, an increase in high deductibles might have diminished the use of expensive tests in fee-for-service plans. Second, the indication for imaging was not available in the data set herein, nor were the results of any risk stratification or D-dimer testing. Therefore, it was not possible to assess whether imaging was appropriate or inappropriate or whether imaging use was associated with improved patient outcomes. Third, we used specific imaging codes to identify CTPA for suspected PE, and some PE studies may have been coded as routine chest CT. Nonetheless, imaging patterns for CTPA paralleled the overall pattern of chest CT, with continued increase in recent years. Therefore, it is unlikely that undercapturing of some examinations performed to screen for PE would have altered the overall conclusions.

## Conclusions

From 2004 to 2016 in 7 US integrated and mixed-model health care systems, rates of chest CT and CTPA performed for suspected PE continued to increase among adults but at a slower pace in more contemporary years. Efforts to combat overuse have not led to a reduction in imaging to screen for PE and at best may have contributed to its slower growth in recent years.
